# The Dynamics of Cytokinin Changes after Grafting of Vegetative Apices on Flowering Rapeseed Plants

**DOI:** 10.3390/plants8040078

**Published:** 2019-03-28

**Authors:** Danuše Tarkowská, Maria Filek, Jan Krekule, Jolanta Biesaga-Kościelniak, Izabela Marciñska, Marzena Popielarska-Konieczna, Miroslav Strnad

**Affiliations:** 1 Laboratory of Growth Regulators, The Czech Academy of Sciences, Institute of Experimental Botany & Palacký University, Šlechtitelů 27, CZ-783 71 Olomouc, Czechia; miroslav.strnad@upol.cz; 2 Department of Biochemistry, Biophysics and Biotechnology, Pedagogical University, Podchorązych 2, 30-084 Krakow, Poland; mariafilek@gmail.com; 3 Institute of Experimental Botany, The Czech Academy of Sciences, Rozvojová 263, CZ-165 02 Prague 6—Lysolaje, Czechia; krekule@ueb.cas.cz; 4 Institute of Plant Physiology, Polish Academy of Sciences, Niezapominajek 21, 30-239 Krakow, Poland; j.koscielniak@ifr-pan.edu.pl (J.B.-K.); i.marcinska@ifr-pan.edu.pl (I.M.); 5 Institute of Botany, Jagiellonian University, Gronostajowa 9, 30-387 Kraków, Poland; m.popielarska-konieczna@uj.edu.pl

**Keywords:** cytokinins, reproductive development, *Brassica napus*, grafting, shoot apical meristem

## Abstract

Despite numerous studies, the role of hormones in the induction of shoot apical meristem leading to reproductive development, especially regarding thermoperiodic plants, is still not fully understood. The key problem is separating the effects of the low temperature required for vernalization from those responsible for low temperature stress. An earlier experiment demonstrated the correlation between an increase of cytokinin level in the apical parts of winter rapeseed and the transition time into their reproductive phase during vernalization, i.e., low temperature treatment. From data obtained from the presented experiments, this study aims to contribute to the understanding the role of cytokinins in the induction of flowering based on the grafting of vegetative apical parts of winter rapeseed (scion) on the reproductive (stock) winter and spring genotypes. On the basis of analyses carried out using ultra-high-performance liquid chromatography coupled with tandem mass spectrometry in combination with microscopic observation of changes at the apical meristem, it was indicated that the increase in the amount of *trans*-zeatin and *trans*- and *cis*-zeatin-*O*-glucoside derivatives appeared in the early stages of apex floral differentiation. During further development, the content of all investigated cytokinins passed through the maximum level followed by their decrease. The final level in reproductive apices was found to be higher than that in vegetative ones.

## 1. Introduction

Flowering is the crucial developmental phase in the lifecycle of seed-bearing vascular plants (angiosperms) and represents their transition from the vegetative to the reproductive stage, i.e., the transformation of the vegetative meristems into inflorescence meristems (IMs) after the plant has passed from the juvenile to the adult phase [1]. IMs then enable a commitment to flowering through the differentiation of its cells to produce floral meristems [2]. The initiation of cell differentiation in the apical meristem associated with achieving the reproductive stage is regulated by a complex of environmental and endogenous inductive cues, such as day length (photoperiodism), temperature (vernalization) and hormones [3,4,5,6]. Among these, cytokinins (CKs) and gibberellins (GAs) are known to be implicated in processes associated with reproductive development in numerous plants [7,8,9,10,11,12]. 

Recently, it has been convincingly demonstrated in *Arabidopsis* that CKs may induce flowering by activating the Flowering Locus T (FT) paralogue twin sister of FT (TSF). This effect possessed the same kinetics as exposure to inductive long days; therefore, CKs should also be considered as an obligatory component of floral induction [13,14]. 

From earlier studies, it was concluded that CKs also participate in the flowering initiation of the thermoperiodic plant *Brassica napus* [15]. The greatest changes (a rise in the CK level) were correlated with the transformation of the shoot apical meristem (SAM), leading to its reproductive development, occurring during the vernalization process, i.e., the period of exposure to cold required for the thermoinduction of flowering. It was suggested that CKs, especially *cis*-zeatin-types (*c*Z-types), were involved in this process. This observation was interesting because there are indications that *c*Z-types function as less active CKs [16]. However, to confirm the role of CKs in the reproductive induction of thermoperiodic plants, it was necessary to separate the effects connected with the action of low temperature from the processes associated with plant abiotic stress where CKs were also found as plant protectors [17,18,19]. This possibility has given rise to experiments of grafting non-vernalized apical parts of shoots on reproductively induced plants [20,21,22]. It was indicated that, after grafting, non-reproductively-initiated apices acquired the capacity to flower. Thus, the aim of this study was to determine the CK content in apical parts of winter rapeseed plants, which were grafted (in the vegetative stage of development, i.e., without vernalization) on reproductive (flowering) plants.

## 2. Results and Discussion

**Figure 1:**
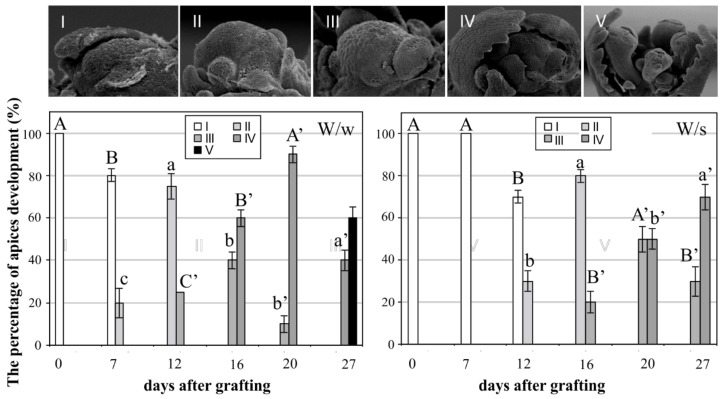
The percentage of apices development of winter rapeseed plants (W) after grafting on flowering winter (w) and spring (s) rapeseed plants. The capital letters (W) indicate flowered stocks and small letters indicate vegetative scions (w, s). Values represent the average ± SE (*n* = 30). Significant differences (*p* ≤ 0.05) between objects at the same developmental phase are marked as different letters according to Duncan’s test.

Microscopic observations indicated that the initiation of reproductive development in vegetative (non-vernalized) winter rapeseed apices occurred independently of the genotype of the plants to which they were grafted (winter, spring), Figure 1. 

However, the stimulation of apices to reproductive development was faster when they were grafted on the winter rapeseed cultivar. After 27 days, about 50% of W/w (winter/winter grafting) plant apices reached the reproductive stage V, while the apices of W/s (winter/spring grafting) plants at the same point in time showed only stage IV of reproductive development (in the amount of 70%, see Figure 1). This “shift in time” of the induction of reproductive development between W/w and W/s required verification as to whether the expected changes in the hormone content appeared at the same development stages in apices.

In the vegetative apices studied, the proportion between analyzed CKs was similar to that reported in earlier work [15], i.e., the highest levels were found for *cis*-zeatin riboside (*c*ZR; about 80% of the amount of other CKs) (data not shown). In the youngest leaves, the total content of all monitored isoprenoid CKs (ISCKs) was lower compared to that found in corresponding apices which they surrounded (~0.070 pmol/fresh weight (FW) for apices and ~0.035 pmol/FW for leaves, respectively). However, trends in their profile were shown to be similar. In spring vegetative apices, which were used for comparison with winter plants, a moderately lower level (~0.050 pmol/mg FW) was detected in the total ISCK content compared to those of winter ones (~0.070 pmol/mg FW). In case of the youngest spring leaves, the total ISCK content was found to be comparable (0.045 pmol/mg FW) with that detected in the youngest winter leaves (0.035 pmol/FW).

**Figure 2:**
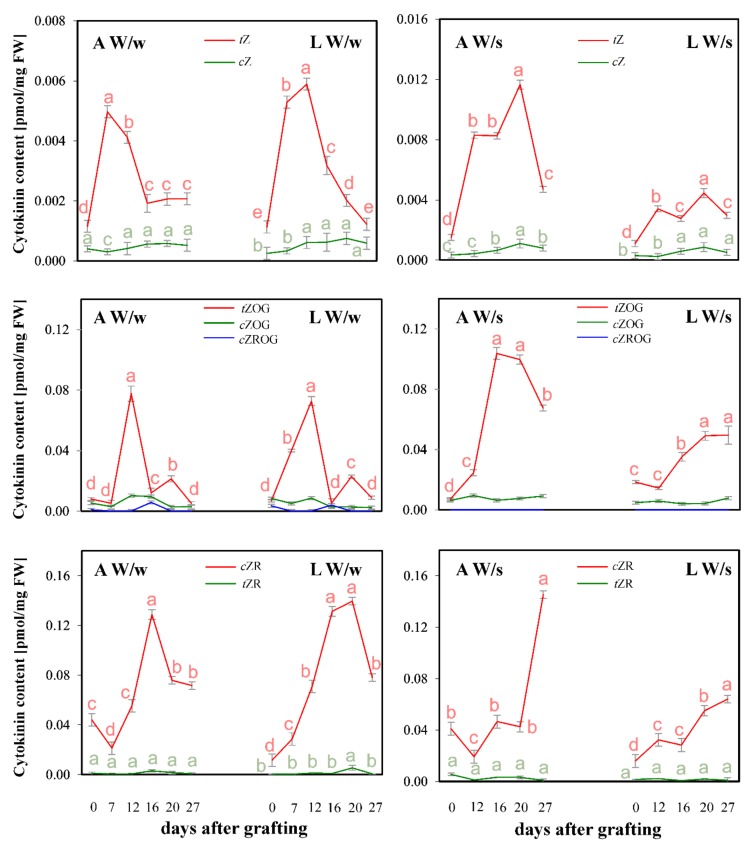
Content (pmol/mg FW) of *cis* (*c*) and *trans* (*t*) zeatin (Z), zeatin *O*-glucoside (ZOG), zeatin riboside *O*-glucoside (ZROG) and zeatin riboside (ZR) in apices (A) and the youngest leaves (L) of the upper part of winter rapeseed plants after 0, 7, 12, 16, 20 and 27 days after grafting on winter, flowering plants (W/w) and after 0, 12, 16, 20 and 27 days after grafting on spring flowering plants (W/s). Significant differences (*p* ≤ 0.05) are marked as different letters according to Duncan’s test.

The induction of reproductive development in apices seemed to correlate with the rapid increase of all investigated CKs. However, the maximum concentration of a variety of CK derivatives occurred at different stages of reproductive initiation in apices, when it was generally observed later in the leaves than in the apices (Figure 2). An increase in biologically active CK *trans*-zeatin (*t*Z) and stable CK storage forms zeatin-*O*-glucosides (both *trans*- and *cis*-form; i.e., *t*ZOG and *c*ZOG) took place as the first symptoms of apex differentiation in W/w plants appeared (7–12 days after grafting, Figure 2). These concentrations decreased when plants reached stage IV of development (i.e., bud formation, 16 days; Figure 1). Confirmation of these CK derivatives participating in the early steps of reproductive development may be the observation that in W/s apices (and leaves) an increase of both zeatin concentrations appeared also relatively early (20 days after grafting). In both W/w and W/s objects analyzed, the content of *c*ZR increased when more than 60% of apices showed stage IV of development (16 and 27 days for W/w and W/s, respectively), Figure 2.

**Figure 3:**
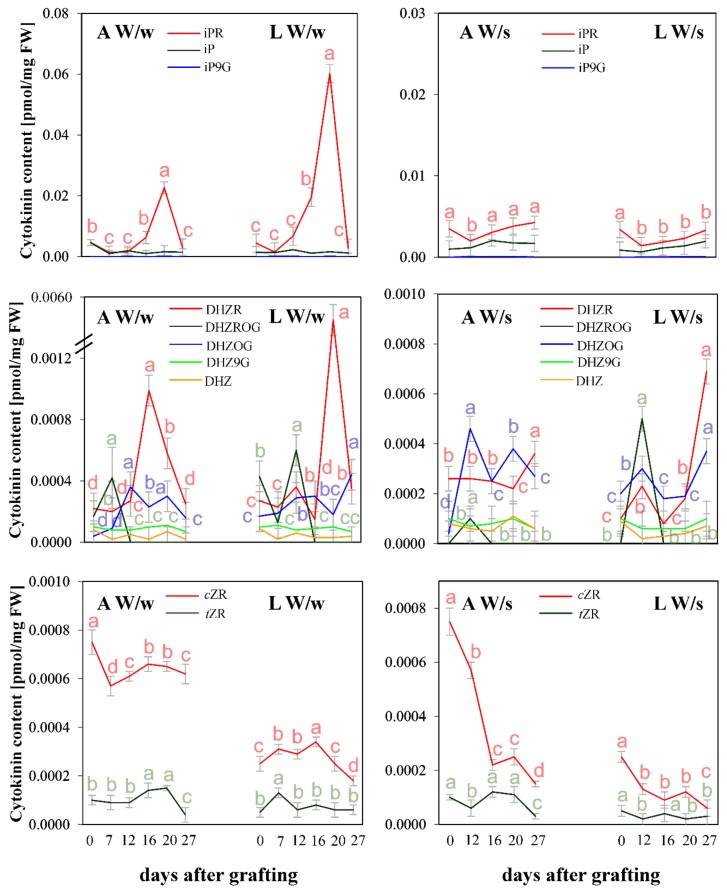
Content (pmol/mg FW) of N^6^-isopentenyladenine (iP), N^6^-isopentenyladenosine (iPR), N^6^-isopentenyladenine-9-glucoside (iP9G), dihydrozeatin (DHZ), dihydrozeatin riboside (DHZR), dihydrozeatin-9-glucoside (DHZ9G), dihydrozeatin *O*-glucoside (DHZOG), dihydrozeatin riboside *O*-glucoside (DHZROG) and *cis* (*c*) and *trans* (*t*) zeatin riboside (*c*ZR, *t*ZR) in apices (A) and the youngest leaves (L) of the upper part of winter rapeseed plants after 0, 7, 12, 16, 20 and 27 days after grafting on winter, flowering plants (W/w) and after 0, 12, 16, 20 and 27 days after grafting on spring flowering plants (W/s). Significant differences (*p* ≤ 0.05) are marked as different letters according to Duncan’s test.

Changes in the content of the other derivatives occurred at a later date (for W/w about 20 days after grafting), Figure 3. For the nucleosides N^6^-isopentenyladenosine (iPR) and dihydrozeatin riboside (DHZR), the rise in their levels was more pronounced in the youngest leaves than in the apices (Figure 3). In W/s plants, the CKs level remained almost unchanged compared to W/w plants except in the levels of biologically inactive CK *c*ZR, where levels reached the maximum before the grafting (day 0) and continually decreased over time with only a small increase 20 days after grafting (Figure 3).

In the reproductive W/w plants, the level of CKs decreased (27 days after grafting). However, their concentrations reached higher values compared to those registered in the vegetative plants. This phenomenon was also shown in a previous study [15]. Thus, the results regarding the changes in CK profiles in both apical parts and leaves may represent markers of floral differentiation of the shoot apices, which appear to be independent of the path of the signals transmission (flowering factors) until needed in their initiation (from winter or spring forms of rapeseed). This fact hinders the use of these data for the evaluation of developmental (vernalization) and stress effects of cold treatment.

## 3. Materials and Methods

### 3.1. Plant Material

The seeds of winter rapeseed (*Brassica nap*. L. var. oleifera, cv. Górczański) after surface sterilization were germinated on blotting paper soaked with distilled water in darkness. Visually uniform seedlings (2 days culture) were transferred into pots filled with a mixture of soil:peat:sand (1:2:1) and grown in a greenhouse with a 16/8 h photoperiod at 20/17 °C and 250 µmol m^−2^s^−1^ irradiance. After 1 month (five-leaf rosette stage), the plants were transferred to a growth chamber and vernalized at 5/2 °C with a 16/8 photoperiod and 250 µmol m^−2^s^−1^ irradiance for 56 days. This is the time required for the full reproductive induction of this genotype [15]. After vernalization, further cultivation (up to the initiation of buds) was performed in a greenhouse at 20/17 °C under the same light conditions as those employed before vernalization. Flowering apical parts were cut off from the plants and replaced by vegetative scions obtained from the apical part of the non-vernalized Górczański plants (about 1.5 cm long, with only its two uppermost leaves). Grafted unions were sealed with Parafilm^®^ tape and the whole graft was closed in a transparent polyethylene bag to ensure a high humidity as described previously [21]. After about 1 week, both reproduction (stocks) and vegetative (scions) parts of the plants were accreted. During further growth, new vegetative leaves developed on the apical parts of the grafted plants, which were then removed. Reproductive leaves, and then flower buds, appeared after about 4–5 weeks of continued culture. 

The apical parts (about 5 mm) and the youngest leaves (two leaves closest to the apex) were collected at 0, 7, 12, 16, 20 and 27 days after grafting (winter/winter grafting, W/w).

In the second experiment, the flowering plants of spring rapeseed (cv. Młochowski) were used as the basis (stocks) for grafting vegetative apical parts (scions) of Górczański plants. The parameters of culture and grafting were the same as described above. The apical parts were collected at 0, 12, 16, 20 and 27 days after grafting (winter/spring grafting, W/s).

The morphological changes from vegetative to reproductive stages which occurred in grafted apices were observed using stereomicroscope and evaluated according to Filek et al. [22] as follows: (I) vegetative rapeseed apices at the five-leaf stage; (II) vegetative rapeseed apices at the six-leaf stage; (III) initiation of reproductive development at the seven-leaf stage, during vernalization, with a vegetative central meristem and the initiation of bud formation; (IV) floral apex at the seven-leaf stage, during vernalization, with formed flower buds; and (V) floral apex at the seven-leaf stage, after vernalization, with formed flower buds. The percentages of apices at subsequent stages of development are presented in Figure 1. For the hormone analysis, samples were frozen in liquid nitrogen and kept at −80 °C. 

### 3.2. Cytokinin Analysis by Ultra-High-Performance Liquid Chromatography-Electrospray Tandem Mass Spectrometry (UHPLC–ESI–MS/MS)

The extraction and analysis of CKs was performed according to Tarkowska et al. [15] with some modifications. Briefly, fresh plant tissue samples of 30 mg fresh weight (FW) were homogenized to a fine consistency with 1 mL of Bieleski solution [23] as the extraction solution. The samples were then extracted overnight after adding CK internal standards (OlChemIm Ltd. Olomouc, Czech Republic). The crude extracts were centrifuged and the corresponding supernatants were further purified using mixed-mode solid phase extraction (SPE) columns. This was followed by ion exchange chromatography combined with reversed-phase chromatography and immunoaffinity chromatography (IAC), using immobilized wide range CK-specific monoclonal antibodies as described by Faiss et al. [24]. Finally, the samples were analyzed by ultra-high-performance chromatography-tandem mass spectrometry (UHPLC-MS/MS; Micromass, Manchester, UK). Quantitation of CKs was performed using the isotope dilution method after processing the MS data by MassLynx™ software (version 4.1, Waters, Manchester, UK). The independent experiments were carried out in two to three replications, where for one replication 30–50 shoot apices and the youngest leaves were collected.
